# Facial Presentation of Crohn’s Disease: Report of a Case

**DOI:** 10.7759/cureus.36024

**Published:** 2023-03-11

**Authors:** Arjavon T Talebzadeh, Nojan Talebzadeh

**Affiliations:** 1 Surgery, California Northstate University College of Medicine, Sacramento, USA; 2 Surgery, South County Surgery Center, San Diego, USA

**Keywords:** face, necrosis, immune system, surgery, infection, crohn's disease

## Abstract

Crohn’s disease is an inflammatory condition of the gastrointestinal system affecting millions of people globally. As clinicians, we are faced with this disease commonly in the lower and middle gastrointestinal tract. The presentation of this condition is rare in the maxillofacial and oral regions. This case report presents a case where the patient presents with an acute severe infection in the head and neck skin region. Familiarity with this case alerts dentists and physicians to look for signs of inflammatory bowel disease as a differential diagnosis in patients with this presentation.

## Introduction

Crohn’s disease is a chronic granulomatous disease that affects the gastrointestinal tract from mouth to anus. There are an estimated 400,000 to 600,000 affected individuals in the United States alone. There is a familial tendency, with siblings of affected individuals having a higher risk to develop this disease [1]. There is no gender preference. The majority of the lesions presentation is in the small intestine and colon. The vast majority of these patients have a symptomatic presentation. Abdominal pain, vomiting, and diarrhea are among the most common presentations. Patients with this disease also tend to lose weight due to the side effects of this disorder. Other ramifications outside of this disease may include arthritis, skin rashes, anemia, a feeling of tiredness, inflammation of the orbits, and lack of the ability to concentrate.

The disease has been associated with the NOD2 gene and its protein [2-5]. This gene and its product allow the host to detect bacterial cell walls. The current understanding suggests that bacteria take advantage of the weak mucosal layer and a decreased ability of bacteria to be cleared from the intestinal wall. Oral involvement of Crohn’s disease occurs in approximately 20% of patients [6]. This could present as the first manifestation of the condition. The presentation could stay intraoral but it may also manifest on the facial skin, a condition known as “orofacial Crohn’s disease.”

## Case presentation

**Figure 1:**
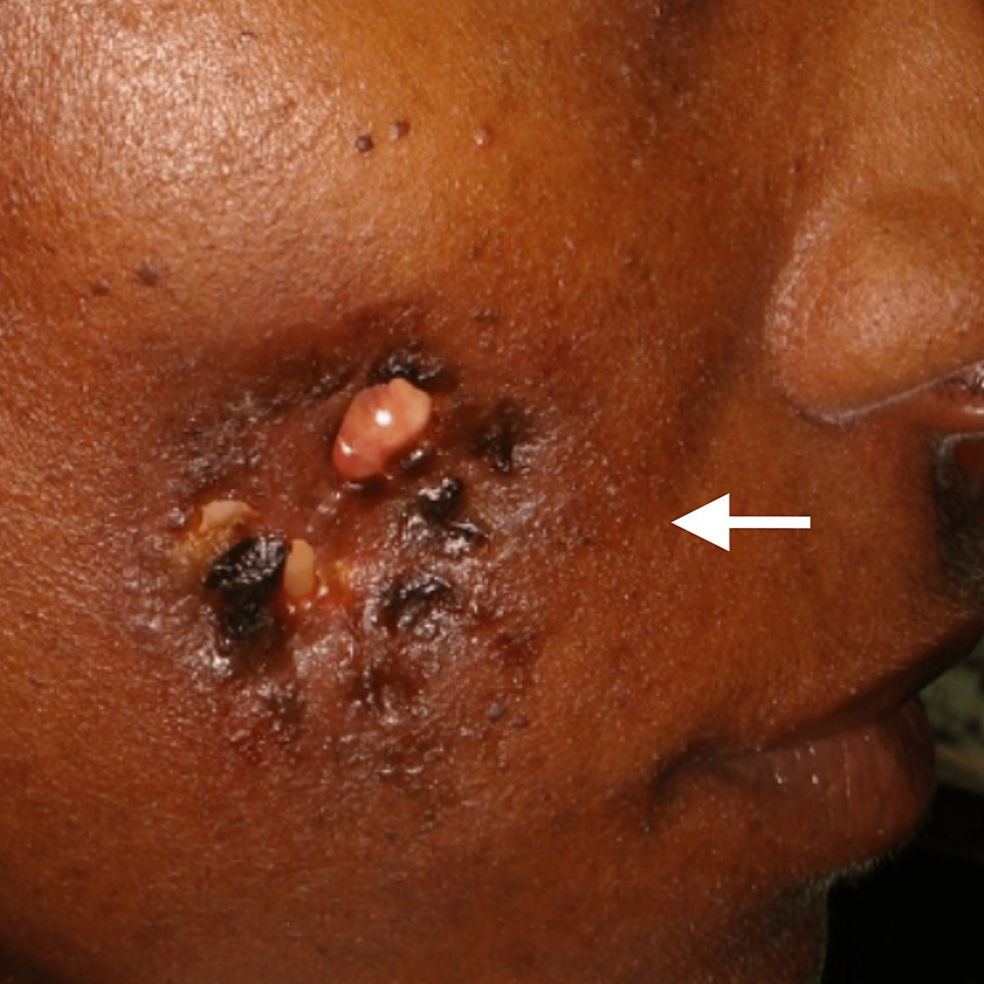
Patient's presentation with a draining fistula and fat extrusion

A 38-year-old African American female presented from the emergency room for the evaluation of a dental infection. She reported a two-month history of fevers and sweating and a dental infection that was worsening over time. The medical history was unremarkable and the patient’s primary physician had ruled out any other medical conditions. The patient appeared to have an ulcer on the facial skin that was not associated with any pain. In the emergency room, the patient's oral exam was significant for one filling missing on the affected site and sensitivity leading to the diagnosis was “dental infection” with a fistula tract extending intra-orally and facially (Figure 1). The patient was sent to the clinic without an X-ray for evaluation.

The patient subsequently presented to her general dentist and the dental evaluation was negative for any dental etiology.

Upon presentation to our office, the examination of her teeth was completely negative. No evidence of dental infection was noticed. The intraoral examination was completely unremarkable with perfect oral hygiene. The facial examination was positive with a large draining abscess. The lesion appeared to have significant extension under the skin with a deep undermining of the dermal layer from the musculature. The patient was subsequently anesthetized and debridement was carried out. Attention was directed to sending cultures and preserving the facial skin.

The differential diagnosis on the list included an infection of a skin cyst (i.e., a sebaceous cyst) and due to the extensive necrosis of skin under the fistula, necrotizing fasciitis was also on differential diagnosis. After culture and biopsy were carried out, the patient was admitted to the hospital for Intravenous antibiotics until possible infection could be controlled.

**Figure 2:**
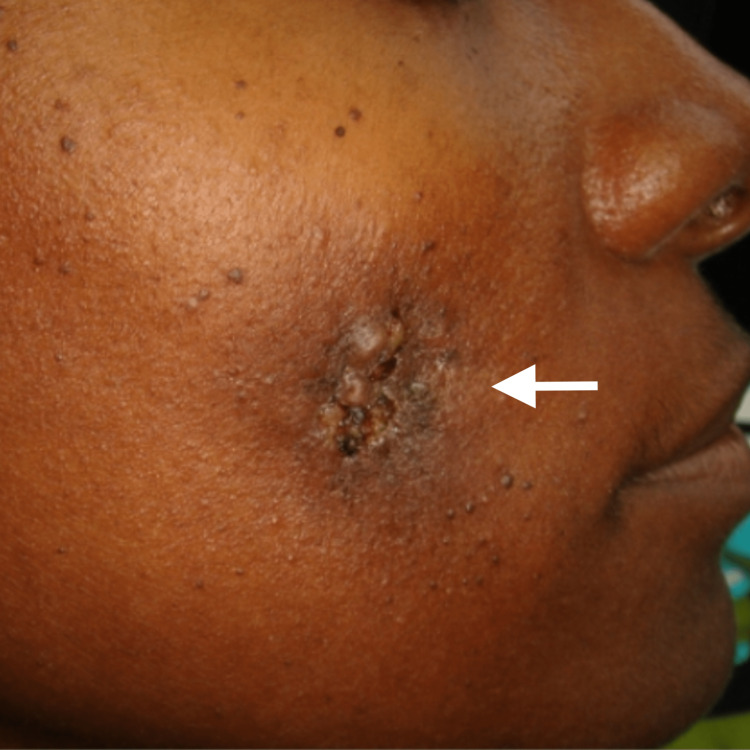
Patient responding to antibiotics and debridement

Immediate culture result presented a Staphylococcus infection and no methicillin-resistant Staphylococcus aureus (MRSA) was detected. Three-day hospital admission resolved the acute infection and the patient was discharged with oral antibiotics for one week (Figure 2).

**Figure 3:**
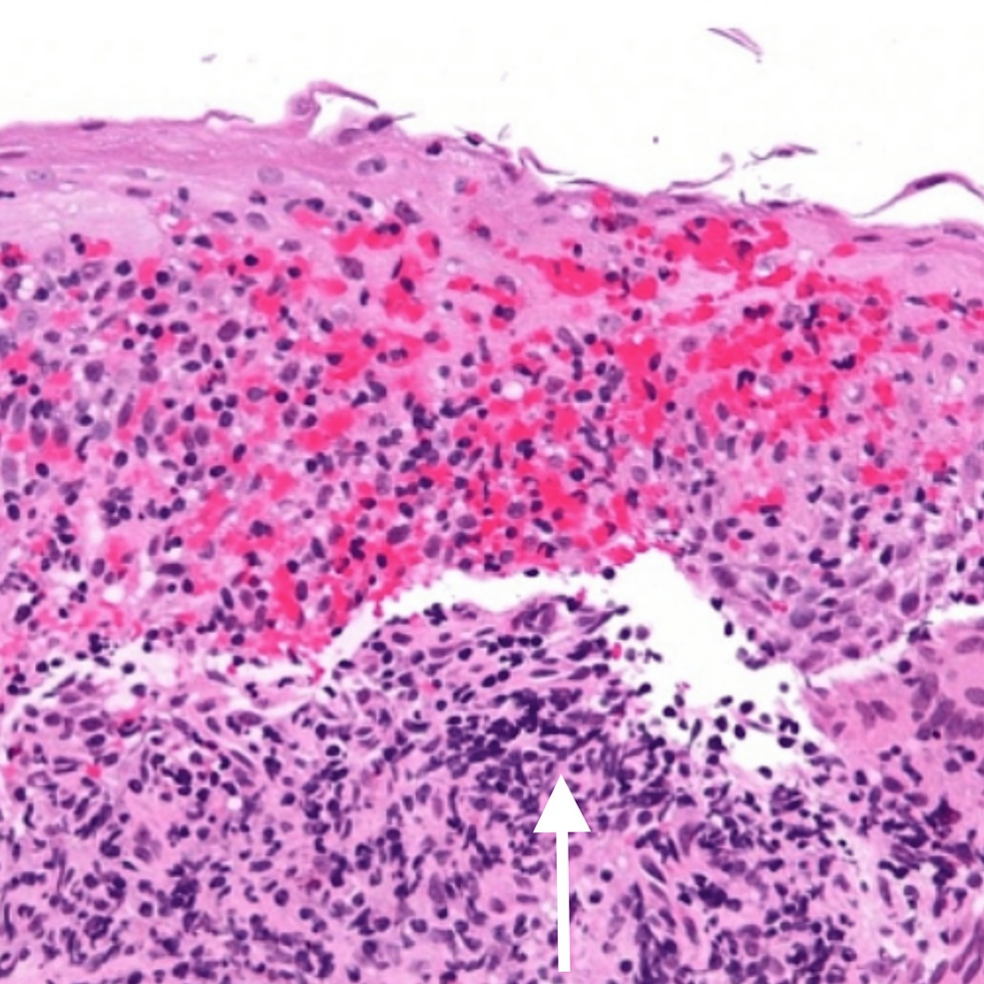
Biopsy of the site with Crohn’s disease

The result of the biopsy was significant for fibrocelluar tissue with evidence of acute and chronic inflammatory cells. An abundance of lymphocytes and macrophages were present. A subepithelial granulomatous lesion was also present (Figure 3).

Based on these findings, the differential diagnosis included orofacial granulomatosis and Crohn's disease with secondary infection. The patient was referred to her primary physician for a workup. After a colonoscopy and endoscopic evaluation, other lesions were noticed and biopsied in the intestinal tract. The results confirmed the diagnosis of Crohn’s disease.

## Discussion

Orofacial Crohn’s disease is not a common condition. Certain ethnicities, such as Jewish heritage (six times more risk) and Europeans (especially Scandinavians) are at a higher risk [7-9]. Patients with a history of inflammatory bowel disease, smoking, living in industrial and urban areas, and eating a lot of sugar and saturated fat had an increased risk of this condition. Other contributors and risk factors include obesity and low sun exposure and low consumption of fruits and vegetables.

Intraorally, it may present as a combination problem with dental infection, which may lead to a missed diagnosis, especially if the patient has poor dental hygiene. The signs of this disease include mucogingivitis, mucosal tags, ulcers, cobble stoning of the lining of the cheeks, and swelling of lips. The patients will have lymphadenopathy of the cervical chain usually on presentation [10,11].

The treatment of the disease includes drug therapy and, in some circumstances, surgery [12-14]. This is a chronic condition that has no cure. The treatments are palliative and attempted by the physicians to manage symptoms. The approach involves a step-up or step-down approach. In step-up, the treatment starts conservatively and is built up. In step-down, the treatment starts aggressively and is reduced over time.

The treatment usually starts with anti-inflammatory drugs such as 5-aminosalicylates and corticosteroids. The aminosalicylates include sulfasalazine (Azulfidine), which contains sulfa, and mesalamine (Asacol, Delzicol, Pentasa, Lialda, and Apriso). These medicines do have side effects such as nausea, diarrhea, and vomiting. The corticosteroids are very effective in reducing inflammation but also have side effects such as moon facies, insomnia, anxiety, hypertension, diabetes, osteoporosis, cataract, and an increased chance of infection. The alternative medication includes immune system suppressors. These drugs include azathioprine (Imuran) and mercaptopurine (Purinethol). These medications are usually used for inflammatory bowel disease. The side effects include liver and pancreatic inflammation and bone marrow suppression. These drugs also increase the risk of infection. Infliximab (Remicade), adalimumab (Humira), and certolizumab pegol (Cimzia) are tumor necrosis factor inhibitors. They are used in children and adults in case other treatment modalities fail. They are provided for severe cases of Crohn’s disease as an alternative to the medication mentioned. Cancer treatment medications, including methotrexate, cyclosporines, and natalizumabs are also used to decrease inflammation and treat fistulas. However, the side effects of these medicines need to be weighed versus the benefits. One of the side effects of Crohn’s disease is infection and fistula formation. These abscesses require antibiotics such as metronidazole and ciprofloxacin to prevent peritoneal infection and spread [15]. Supplemental medication, such as anti-diarrheals, pain relievers, iron supplements, and multivitamins can also relieve the side effects of this condition.

Finally, surgery is the treatment if the disease does not react positively to medication. The surgeon will remove the portion of the gastrointestinal tract that does not respond to medication and reconnect the bowel. However, in the face and oral cavity, the luxury of the resection is less likely. The resection of facial skin could lead to a significant amount of deformity. In our patient, the lesion had extended to the surface and the surgical approach involved the removal of the infected and necrotic skin and subcutaneous skin with the packing of the wound to allow granulation and gradual closure of the facial skin fistula. This patient opted not to have any cosmetic procedures done but facial cosmetic procedures such as Fraxel laser resurfacing combined with chemical peels and micro-needling could be used to minimize the scar and the disfiguring of the facial skin.

## Conclusions

There are various presentations of Crohn’s disease. Patients may have any of the symptoms mentioned above or any combination of those could be a presentation. A practitioner should be aware of the possible presenting symptoms and be able to manage them accordingly. It is important to realize that usual patients can be responsive to some medications and not to others and combination treatment is very common. In this patient, the presentation was highly unusual due to the disease presenting on the skin side of the face and not an extensive intra-oral component. the patient responded well and is presently managed in a gastrointestinal clinic.
